# Research Progress of Cell Lineage Tracing and Single-Cell Sequencing Technology in Malignant Skin Tumors

**DOI:** 10.3389/fsurg.2022.934828

**Published:** 2022-06-16

**Authors:** Ang Li, Baoyi Liu, Jingkai Xu, Yong Cui

**Affiliations:** ^1^ Department of Dermatology, China-Japan Friendship Hospital, Beijing, China; ^2^ Graduate School, Peking Union Medical College and Chinese Academy of Medical Sciences, Beijing, China

**Keywords:** cell lineage tracing, single-cell sequencing, malignant melanoma, skin tumor, cutaneous squamous cell carcinoma (cSCC)

## Abstract

Cell lineage tracing and single-cell sequencing have been widely applied in development biology and oncology to reveal the molecular mechanisms in multiple basic biological processes and the differentiation of stem cells, as well as quantify the differences between single cells. They provide new methods for in-depth understanding of the origin of tumors, the heterogeneity of tumor cells, and the drug resistance mechanism of tumors, thus inspiring new strategies for tumor treatment. In this review, we summarized the progress of cell lineage tracing technology and single-cell sequencing technology in the research of malignant melanoma and cutaneous squamous cell carcinoma, attempting to spark new ideas for further research on skin tumors.

## Introduction

Cutaneous malignant melanoma (MM) and cutaneous squamous cell carcinoma (cSCC) account for a high proportion of malignant skin diseases and threaten lives. Although traditional high-throughput sequencing techniques have uncovered many disease-causing or mutated genes, cellular heterogeneity is a hallmark of tumors, and sequencing results at the average level of a population of cells mask expression differences between individual cells, thereby limiting our understanding of cancer. As technology advances, cell lineage tracing and single-cell sequencing have significantly improved explanation of tumorigenesis and pathogenesis. Among the skin malignancies, MM and SCC have higher malignancy and extensive research bases. Therefore, we reviewed the application of cell lineage tracing and single-cell sequencing technology in them.

### Cell Lineage Tracing

**Figure 1:**
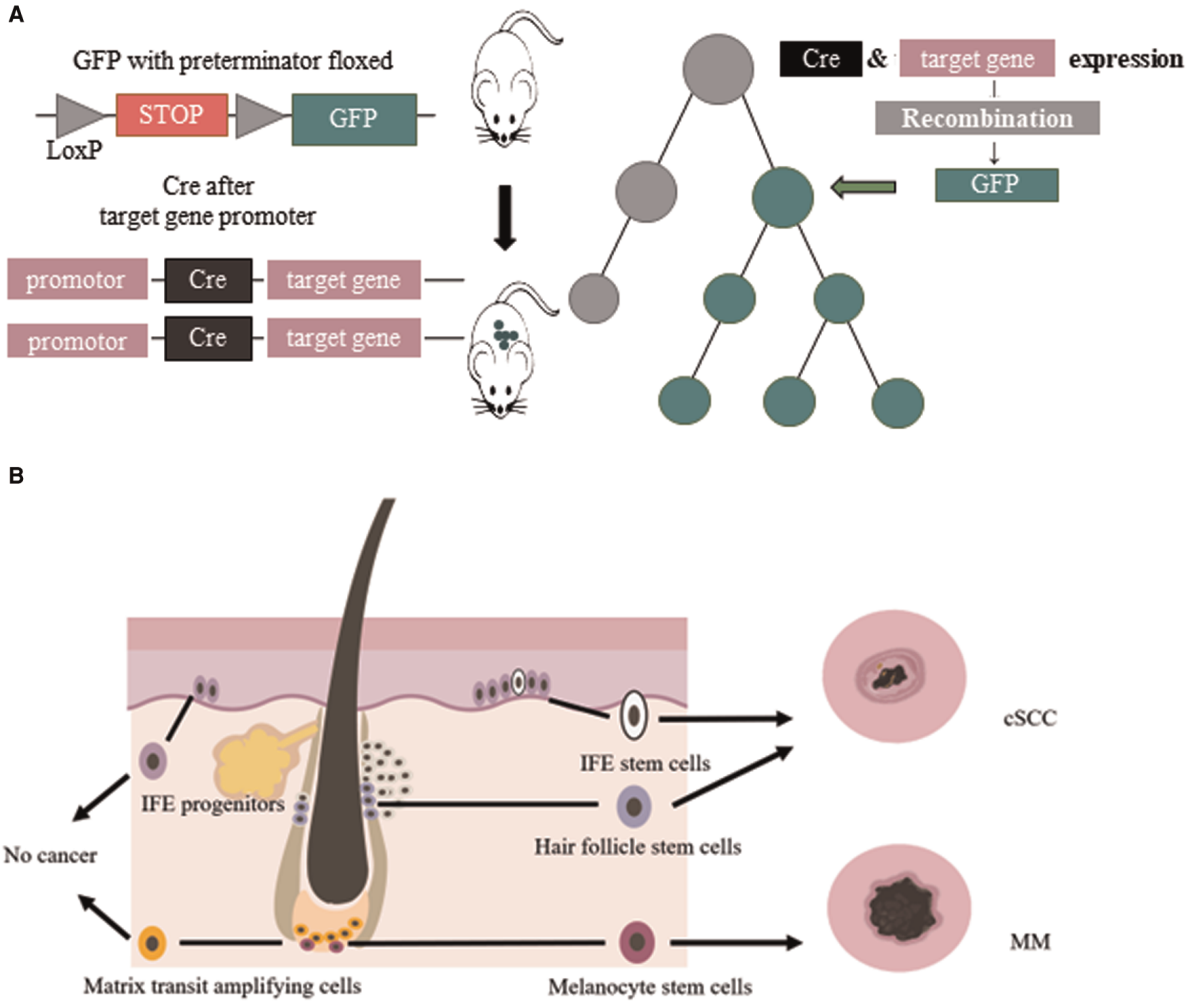
(**A**) Cre/LoxP system for lineage tracing: Transgenic animals with a green fluorescent protein (GFP) gene preceded by a floxed terminator, and a recombinase gene added in front of a gene specifically expressed in the target cell of interest were used. When the cell type begins to show its differentiated characteristics, the recombinase is simultaneously expressed, followed by the expression of GFP. (**B**) Cellular origins of MM and cSCC: IFE stem cells and HFSCs could develop in to cSCC. MCSCs located at the base of hair follicles could develop in to melanoma by external stimulation. Other cells, such ad IFE progenitors, do not become cancerous.

Multicellular organisms proliferate, divide and differentiate from a single cell, and eventually form an individual with distinct organization and different morphology. This developmental process is a lineage. Cell lineage tracing refers to the use of various methods to mark a cell, and to track and observe the proliferation, differentiation and migration of all its descendants to reveal the transition of cells from one type to another, or from one state to another, through the construction of cell trajectories and gene expression changes along the timeline (1). Lineage tracing provides an important means to study organ development, tissue damage repair, and single-cell differentiation fate. Breakthroughs in cell lineage tracing technology, especially the inducible recombinase Cre/LoxP system, have expanded the application of cell lineage tracing technology (2) (Figure 1).

### Single-Cell Sequencing

Single-cell sequencing refers to the amplification and sequencing of the transcriptome or genome at the single-cell level. According to the differences in sequencing molecules, single-cell sequencing mainly includes single-cell genome sequencing, transcriptome sequencing, immunology sequencing, copy number sequencing, transposase and nuclear chromatin sequencing, as well as single-cell surface protein detection. Single-cell sequencing has been applied in the fields of reproduction, immunity, stem cell differentiation, embryonic development, and tumor heterogeneity (3). Since the first single-cell transcriptome sequencing technology was available in 2009, the emergence of multiple single-cell sequencing platforms such as Fluidigm, WaferGen, 10× Genomics, inDrop, Drpo-seq, and Illumina/Bio-Rad has greatly promoted life sciences (4–7). Lineage tracing combined with single-cell sequencing technology has begun to decipher cell fate and gene expression, providing new ideas for research on skin tumor pathogenesis, drug development, and personalized medication (8, 9).

### Cutaneous Malignant Melanoma

MM is the deadliest skin cancer. The latest global cancer statistics show that in 2020, there were 324,635 new cases of cutaneous melanoma and 57,043 deaths (10). Cutaneous MM is caused by the malignant transformation of melanocytes located at the base of the epidermis, and 30% of cutaneous MM evolve from pigmented nevus. The most commonly mutated genes in cutaneous MM include *BRAF*, *PTEN*, *NRAS*, *TP53*, *CDKN2A*, *MITF*, and *BAP1* (11–13). *BRAF* mutations were discovered in about 50% of all cutaneous MM patients, about 40% of whom also carried *PTEN* mutations (12).

### Cell Lineage Tracing in MM

Lineage tracing has made valuable contributions to studying the relationship between melanocyte stem cells (MCSCs) and MM. Murine MCSCs are located in the hair follicles of the dorsal and ventral skin, both MCSCs and hair follicle stem cells undergo cyclical periods of rest (telogen) and activation (anagen) that define the hair cycle. MCSCs could proliferated to those MCSCs that do not produce melanin and mature melanocytes. Tyrosinase (Tyr) is an important molecular marker of MCSCs (14). Lineage tracing revealed that MCSCs are one of the origins of cutaneous MM, and Tyr-CreER^T2^; BRaf^V600E^; Pten^f/f^ mice model was widely used in study MM (15–18). Kohler et al (17) identified that cutaneous MM could originate from bulge melanocytes but only after these cells have migrated out of the bulge niche and reached the lower HF region as mature melanogenic melanocytes. Moon et al. (18) identified that during melanoma development, quiescent MCSCs resists to tumorigenesis, and mature pigment-producing melanocytes might induce tumorigenesis (Figure 1). In the follicle cycle, the hair follicles that changing from telogen to anagen were more likely to induce tumors, while those that experiencing anagen to telogen did not present this feature. Quiescent hair follicles did not induce melanoma, but external stimuli such as UV light could activate MCSCs and lead to cutaneous MM (17). In addition, the clever combination of traditional lineage tracing and other techniques provided more precise origin tracing. Rewind, a striking new technique has been developed by Benjamin et al. (19) Combining genetic barcodes with RNA fluorescence in situ hybridization, it can directly capture rare cells in primitive subpopulations. Its application in MM directly captured drug-resistant precursor cells from V600E melanoma cells, allowing researchers to unearth some of the characteristics of such cells. In conclusion, tracing the origin of MM is of great significance for guiding and validating potential treatment options.

### Single-Cell Sequencing in Cutaneous MM

Single-cell sequencing has been widely used in studying MM, including single-cell transcriptome sequencing, single-cell exome sequencing, and single-cell immunomic sequencing (20). Tirosh et al. (21) applied single-cell transcriptome sequencing for the first time in 19 melanoma patients, analyzed the cell types of the tumor tissue, and elaborated on the expression pattern of immune cells in the tumor. The study also unveiled significant cellular heterogeneity in different regions of the tumor tissue. Analysis of tumor-associated fibroblast populations revealed that they influenced the presence of specific cellular signals and melanoma drug resistance. Since then, a multi-omics map of single-cell sequencing MM has been constructed.

Drug resistance often results in tumor recurrence, increasing financial pressure on patients, and the likelihood of death. To this end, more single-cell sequencing studies were devoted to drug resistance mechanisms in cutaneous MM (22–25). Single-cell sequencing of different MM tissues and comparison of their heterogeneity revealed the characteristics of many tumor cells and associated immune cells. An earlier study using single-cell transcriptome sequencing to analyze the cutaneous melanoma tissue of 33 human cases revealed that a tumor’s T-cell signature could classify most tumors as those with a T cell exclusion program. Exclusion program was applied to samples treated with ipilimumab and anti-programmed cell death protein 1 (PD1) antibody to study its role in drug-resistant tumors (23). Single-cell transcriptome sequencing of CD8+ T cells in peripheral blood of metastatic melanoma patients who received anti-PD1 therapy revealed that the proportion of mucosa-associated invariant T cells was increased in responders (26). A Smart-seq2 study performed single-cell transcriptome sequencing of 48 tumor samples taken from biopsies of 32 patients with metastatic melanoma. In three groups of patients treated with PD1 inhibitor, anti-CTLA-4, and combination therapy, CD8+ T cells were found to be a key component of the immune checkpoint inhibitory mechanism, which is abundant in melanoma and closely related to tumor depletion. The CD8+ T cell transcription factor TCF7 can be used to assess the prognosis of immunotherapy (22). In addition, Pauken et al. (27) found melanoma-associated TM (tumor-matching CD8+ T cells, marked as NKG2D, CD39, and CX3CR1) cells in the peripheral blood of humans and mice, which also suggests that monitoring of T cells has a considerable therapeutic potential.

**Figure 2:**
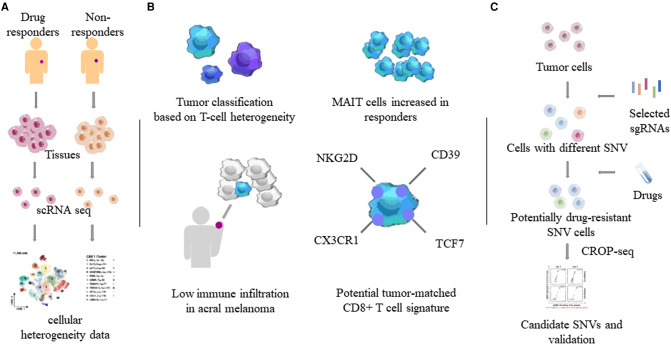
Heterogeneity of tumor and immune cells by single-cell sequencing. Through single-cell sequencing of different tumor tissues and related tissues such as blood, the characteristics of different tumors and tumor-related immune cells, especially T cells, were obtained. (**A**) Determination of tumor heterogeneity using single-cell sequencing. (**B**) T cell properties in MM. MAIT: mucosa-associated invariant T cells. (**C**) One combination of the CRISPR/Cas system and single-cell sequencing. SNVs: single nucleotide variants.

Combining CRISPR RNA-guided deaminase and CRISPR droplet sequencing, a study incorporated NRAS, KRAS, and MAP2K1 in the MAPK pathway of A375 melanoma cells and transferred a total of 420 sgRNAs designed for these three genes. In order to monitor the drug response to BRAF inhibitors in melanoma cells, multiple drug resistance mechanisms related to the targeted therapy of cutaneous melanoma have been discovered (25). Perturb-CITE-seq read out the single-cell transcriptome and proteome in the interaction of CRISPR-Cas9 with immune checkpoint inhibitors in melanoma cells, uncovering potential clinically relevant immune evasion mechanisms (28). Minimal residual disease (MRD) can lead to recurrence after melanoma treatment. Xenograft melanomas from BRAF-mutated patients were treated with RAF/MEK inhibition followed by the induction of an MRD model. Distinct cell subsets identified by scRNA-seq revealed that the drug-resistant state of cells within MRDs could be driven by adaptive, non-mutational events independent of mutation (29). Compared to non-acral cutaneous melanomas, acral melanoma had a lower immune infiltrate with fewer effector CD8 T cells and NK cells and a near-complete absence of γδ T cells (30) (Figure 2).

These single-cell sequencing studies have uncovered heterogeneous characteristics of tumors and associated immune cells, revealing mechanisms of immune evasion that are extremely beneficial in the search for novel therapies.

### Cutaneous Squamous Cell Carcinoma

Cutaneous squamous cell carcinoma (cSCC) is the second most common non-melanoma tumor among skin malignancies, following only basal cell carcinoma (31). Mutated genes associated with cSCC include *TP53*, *HRAS*, *NRAS*, *KRAS*, *CDKN2A*, *NOTCH1*, *NOTCH2*, *PIK3CA*, *TGFβ*, *PTEN*, *BRAF*, *EGFR*, etc (32). Cell lineage tracing and multiple single-cell omics studies have contributed to the tumor origin and etiology of cSCC.

#### Cell Lineage Tracing in cSCC

The tumor origin theory is a major focus of current research and has important implications for the etiology of tumors. The development of single-cell lineage tracing has facilitated the traceability of cutaneous SCC. Studies have shown that cSCC could originate from interfollicular epidermis (IFE) stem cells and hair follicle stem cells (HFSCs), but not from hair follicle transient amplifying cells (TACs) or epidermal IFE precursor cells, and the malignant degree of cutaneous SCC tumors of different origins varies (33–36). KRas^G12D^ mutation activation and Tp53 deletion are the most common gene mutations in cSCC. K5 and K14 are marks of IFE, HFSCs have K19, Lgr5 and Sox9 as marks, and shh can mark TACs. Cross-specific driver mice were crossed with Kras^G12D^ mutant-activated mice and Tp53-null mice. In addition, cutaneous SCCs derived from HFSCs were prone to epithelial mesenchymal transition (EMT) and more invasive (36) (Figure 1). In K14-creER; KRas^G12D^; Tp53^f/f^ mice, the mesenchymal marker Vimentin of IFE stem cell-derived highly differentiated SCCs was restricted to the tumor mesenchymal region, and the expression of epidermal markers, epithelial cell adhesion molecule (Epcam) and E-cadherin, increased. However, the SCCs derived from HFSCs of Lgr5-creER; KRas^G12D^; p53^f/f^ mice expressed Vimentin in all intertumoral tissues, the expression of Epcam and E-cadherin decreased, and the tumors were more prone to EMT and had a higher malignancy (36, 37).

#### Single-Cell Sequencing in cSCC

Sequencing studies at the whole-cell level, such as transcriptome sequencing and chromatin accessibility sequencing, have found some potential targets for tumor therapy, but there are still huge challenges in the observation of tumor heterogeneity (36, 38). By analyzing whole transcriptome profiles at single-cell resolution, the heterogeneity of tumor cells was revealed. Annotating different cell types from tumor tissues, genes belonging to the S100 gene family, SPRR gene family, and FABP5 are highly expressed in cSCC cells, and these pathways may be targets for future disease treatments (39).

The immune status of T cells is associated with the pathogenesis of SCC. In 2020, Ji et al. (40) used a combination of single-cell RNA-seq, spatial transcriptome sequencing, multiplex ion beam imaging, CRISPR screening and other methods to comprehensively analyze and study cSCC. The study found four tumor cell subsets, three of which covered the normal epidermal state, and one formed by tumor-specific keratinocytes was localized in the fibrovascular microenvironment. Multiple potential immunosuppressive features, including co-localization of Tregs with CD8 cells in the compartmentalized tumor stroma, were confirmed. Furthermore, they identified the important role of specific tumor subpopulation-enriched gene networks in tumorigenesis through single-cell characterization of tumor cell xenografts in humans and in vivo CRISPR screening. The study clarified the spatial niche of the interaction between cSCC and stromal cell subsets and the gene network involved in cancer, which provides a basis for the study of the mechanism and treatment of cSCC. Single-cell TCR sequencing of cSCC on immunosuppressed patients receiving organ transplantation and on patients with normal immunity showed that transplant-associated SCC had a higher regulatory T cell/cytotoxic T cell ratio, which facilitated its evasion in a special immune microenvironment (41). An scRNA-seq-based study on the cSCC mouse model found that tumor-initiating stem cells selectively acquired CD80 and could directly inhibit the activity of cytotoxic T cells, suggesting an important role in activating immune checkpoint therapy (42). scRNA-seq was performed on cSCC patients before and after anti-PD-1 treatment, and it was found that pre-existing tumor-specific T cells may have limited reinvigoration capacity, and that T cell responses to checkpoint blockade were dependent on the recruitment of novel T cells (43). CD276 is highly expressed on the surface of head and neck SCCs and acts as an immune checkpoint to allow cancer stem cells to evade surveillance by the immune system. Murine models combined with single-cell sequencing showed that blocking CD276 reduced epithelial-mesenchymal transition (44).

Tumor evasion characteristics and immune cell status discovered by single-cell sequencing studies surrounding tumor tissue provide direction for targeted therapy.

## Summary

With the advent of cell lineage tracing and single-cell sequencing, the origins and development of many diseases have been gradually uncovered. In tumor research, lineage tracing reveals the origin and clonal evolution of tumor cells, providing novel tumor therapeutic strategies targeting tumor stem cells. And single-cell sequencing is pivotal for understanding tumor heterogeneity, tumor microenvironment, and tumor resistance mechanisms in immunotherapy (45). Although, the application of these two techniques in skin tumors is limited by high cost, equipment, and cell capture requirements, etc., they still breed more attractive and promising research.
